# Investigating the Contribution of Blending on the Dough Rheology of Roller-Milled Hard Red Wheat

**DOI:** 10.3390/foods12102078

**Published:** 2023-05-22

**Authors:** Anu Suprabha Raj, M. Hikmet Boyacioglu, Hulya Dogan, Kaliramesh Siliveru

**Affiliations:** 1 Department of Grain Science and Industry, Kansas State University, Manhattan, KS 66506, USA; asuprabharaj@ksu.edu (A.S.R.); hboyacioglu@kpmanalytics.com (M.H.B.); dogan@ksu.edu (H.D.); 2 KPM Analytics, Westborough, MA 01581, USA

**Keywords:** blending, damaged starch, dough rheology, flour functionality, particle size, tempering

## Abstract

The flour functionality and particle size distribution of wheat flour obtained on roller milling are dependent on the type of wheat, tempering conditions, and milling conditions. In this study, the impact of the tempering conditions (moisture and time) on the chemical and rheological properties of flour from blends of hard red wheat were analyzed. The wheat blends B1-25:75 (hard red spring (HRS)/hard red winter (HRW)), B2-50:50, and B3-75:25, which were tempered to 14%, 16%, and 18% for 16, 20, and 24 h, respectively, were milled using a laboratory-scale roller mill (Buhler MLU-202). Protein, damaged starch, and particle characteristics were influenced by blending, tempering, and milling streams. For all the blends, the protein content varied significantly among the break flour streams; the damaged starch content varied greatly in the reduction streams. The increased damaged starch content of the reduction streams proportionally increased water absorption (WA). Higher proportions of HRS in the blends significantly decreased the pasting temperature of the dough, as measured using Mixolab. Principal component analysis proved that the protein content was the key determinant in particle characteristics, WA, and pasting properties of the flour, especially in blends with a higher proportion of HRS.

## 1. Introduction

Nearly 20% of humans’ calorie intake worldwide is dependent on wheat [1]. Grain is consumed globally in different forms, such as leavened and unleavened bread, cakes, cookies, and pasta. Wheat flour is the main ingredient in these products. Therefore, it is necessary to recover flour of desirable processing quality. The process involved in the extraction of flour from kernels is termed as wheat milling. During milling, the starchy endosperms of wheat kernels are separated from their outer layers (bran) and are reduced to flour. The factors contributing to efficient separation depend on the kernel quality and processing conditions. Flour quality is determined to a great extent by the type of wheat; however, the processing conditions (mill settings and tempering conditions) can also influence the physicochemical properties and particle characteristics of the flour [2,3,4]. For instance, the setup and environmental conditions of the mill can determine up to 25% of the functionality of flour; the remaining 75% is dependent on wheat quality [5].

According to Campbell [4], the two key tools for ensuring consistent flour quality are wheat tempering and blending. Tempering is the process of conditioning wheat kernels for milling through the controlled addition of water followed by a resting period. The process is essential to exaggerate the friability differences between endosperm, germ, and bran [5]. This mellows the endosperm and toughens the bran of wheat kernel to increase the efficiency of milling [6]. The factors influencing the rate of moisture uptake by wheat kernels are the kernel properties, temperature, tempering time, and initial moisture content of wheat [6,7]. The flour extraction rate, composition, and functionality are altered by the tempering conditions. For instance, the flour yield, ash content, and functionality of soft wheat flour can be modified by increasing the tempering time from 3 to 24 h [7]. Similarly, tempering moisture alters the granulometric composition of flour [8] and the rate of flour extraction [9], and influences energy consumption during the milling process [10].

In general, the quality of wheat is determined to a great extent by the environment and management practices [11,12,13,14]. These factors result in wheat (of any variety) exhibiting a range of quality characteristics. Variations in quality even within the same variety compel millers to resort to blending to ensure consistent quality. Blending is defined as the process of mixing two or more wheat varieties or classes in a given ratio to obtain a wheat mixture that yields desired flour functionality after milling [15]. This process is necessary to ensure consistency in flour quality and cost optimization [15,16]. In practice, wheat of different qualities is mixed during blending to obtain flour of desirable quality [17]. The wheat varieties chosen for blending might vary in physical properties, not simply in chemical composition. This can affect the granulometric composition of the flour, and therefore the quality. Mostly, the published literature focuses on optimizing blending in terms of the final flour quality and cost of operation [16,17,18]. Additionally, wide variability exists in the composition and rheological properties of flour obtained from different milling streams [19,20,21]. This also increases the possibility of modifying the final product quality by blending flour from different streams. Hence, the final flour quality can be altered by blending, modifying the tempering conditions, and adjusting mill operational parameters such as the roll gap, roll speed and roll differential. Thus, the blending process plays a significant role in the wheat supply chain and is vital in ensuring the profitability of the milling process [16].

Although there have been numerous studies on the impact of tempering on flour quality and biochemical variations in milling streams, studies on the impact of blending on the composition and rheological parameters of milling streams are limited. Additionally, the published literature on blending focuses on blending wheat varieties of the same cultivars. For instance, the authors of [22] detailed how to improve the functionality of pest-damaged wheat by blending it with undamaged wheat of the same variety, while in [23], wheat blends of the same cultivar were harvested at different maturities. Moreover, the authors studied the effect of the blending ratio on the physicochemical and rheological characteristics of straight-grade flour (i.e., a homogeneous mixture obtained by combining flour from all milling streams). Nevertheless, the influence of blending on the functionality of flour from different milling streams is often overlooked, as are the tempering conditions. Understanding the compositional variations in different milling streams as a function of blending and tempering would assist in optimizing the milling process of hard wheat, both in terms of quality and cost. Furthermore, the global market for bread is expected to increase to 135 million tons by 2025 [24]. Subsequent rises are evident in the consumption and retail market price of bread [24]. This necessitates further studies on hard wheat blending and its impact on flour functionality. Thus, the specific objective of this study was to understand the influence of blending different cultivars of wheat (i.e., hard red winter and hard red spring) on the composition, particle characteristics, and functionality of flour from different milling streams. Moreover, the impact of tempering conditions (i.e., moisture and time) on the chemical and rheological properties of flour from the blends were studied.

## 2. Materials and Methods

### 2.1. Test Material

The hard red winter (HRW) and hard red spring (HRS) wheat grains used in the study were procured from Montana Flour and Grain (Fort Benton, MT, USA). The moisture contents of the grains were measured following ASABE Standard S352.2 for whole kernels using the oven method [25]; protein contents were assessed using the AACC 46-30.01 protocol [26]. The moisture and protein contents of the HRW wheat grain were 13.2% (w.b) and 10.5%, respectively, whereas those of the HRS wheat grains were 11.7% (w.b) and 16.5%, respectively.

### 2.2. Milling

**Figure 1:**
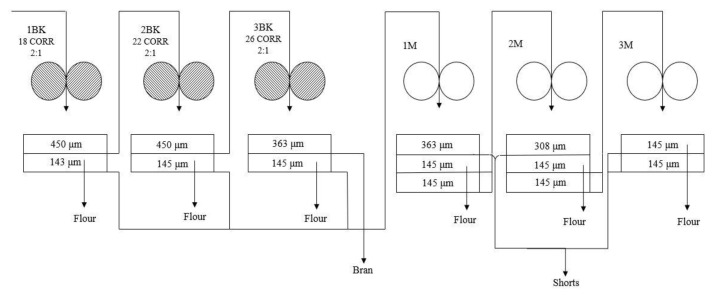
Mill flow sheet of the laboratory mill (Buhler MLU-202). BK: break roll, M: reduction roll, CORR: corrugations per inch on the roll (e.g., 18 CORR-18 corrugations/inch); 2:1 is the roll differential.

Initially, the wheat samples (HRW and HRS) were tempered under the studied conditions (i.e., tempering time of 16, 20, and 24 h and moisture content of 14%, 16%, and 18%). Thereafter, the tempered HRW and HRS wheat samples were mixed in different ratios to obtain blends (2000 g) of varying protein content. These blends were milled using a laboratory mill (Model: MLU 202, Make: Buhler Bros, Switzerland) according to AACC 26-21.02 [26]. The three different blends used in the study were B1 (25% HRW:75% HRS), B2 (50% HRW:50% HRS), and B3 (75% HRW:25% HRS). After milling, the flour from three break rolls (1 BK, 2 BK, and 3 BK) and reduction rolls (1 M, 2 M, and 3 M) was collected separately for all wheat samples (Figure 1). Furthermore, the straight-grade flour was prepared by recombining the flour from all the streams. For the experiments, all other milling parameters, including roll gap and roll differential, were kept constant.

### 2.3. Compositional Analysis

The chemical composition of the flour streams and straight-grade flour was determined according to standard protocols: crude protein (AACC 46-30.01), ash (AACC 08-01.01), moisture content (AACC 44-15.02), and damaged starch (AACC 76-33.01) [26]. The amperometric method of damage starch analysis using SDmatic (Model: SDmatic, Make: Chopin technologies, Villeneuve-la-Garenne, France) is based on the absorption of iodine by damaged starch. Briefly, a solution was prepared by adding 120 mL of distilled water containing 3 g of potassium iodide (KI), 1.5 g of citric acid (C_6_H_8_O_7_), and 1 drop of sodium thiosulphate (Na_2_O_3_S_2_). After the solution in the reaction bowl reached 35 °C, 1 g of flour was dispersed into the solution. The absorption of iodine by starch varied with the intensity of the damage and was measured.

### 2.4. Particle Size Analysis

The particle analyzer Malvern Morphology G3 (Model: G3, Make: Malvern Panalytical, Grovewood Road, UK) was used to determine the size distribution of the flour samples. The flour sample (5 mm^3^)was transferred to the dispersion chamber of the particle size analyzer, where it was dispersed as a thin layer on the glass slide [27]. Subsequently, the equipment analyzed the size characteristics of 5000 particles in a predefined area within the dispersion [28]. The size distribution parameters obtained from the measurement included *d10*, *d50*, and *d90*. The values designated as *d10*, *d50*, and *d90* indicate that 10, 50, and 90% of the measured particles are smaller than their respective value. It should be noted that all these diameters were calculated based on the assumption that the particles are spherical.

### 2.5. Thermomechanical Characteristics

**Figure 2:**
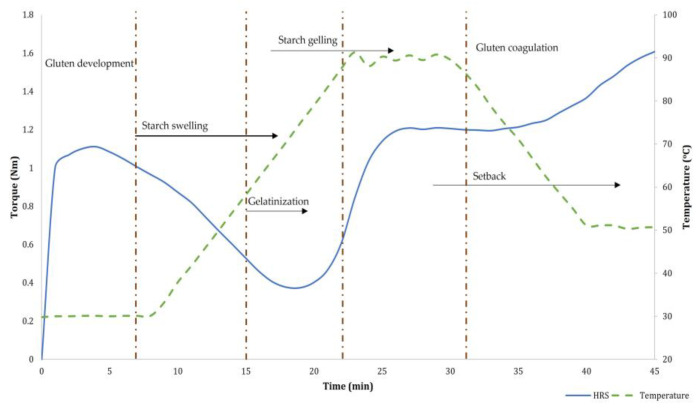
Sample Mixolab profile representing the changes during the Mixolab analysis.

The variations in the consistency of dough, when subjected to mixing with a gradual increase in temperature, were measured using the instrument Mixolab. Mixolab analysis has the potential to simultaneously assess the rheological properties and the viscoamylograph characteristics of the dough subjected to heating and cooling cycles (viscoamylograph measurements are comparable to measurements from the rapid visco analyzer) [29]. Moreover, the equipment measures the water absorption capacity, dough stability during mixing, gelatinization characteristics, and retrogradation of starch in a single analysis [30]. Accordingly, the rheological behavior of dough as a function of mixing and temperature was studied following AACC 54-60.01 [26] using Mixolab (Model: Mixolab, Make: Chopin Technologies, Villeneuve-la-Garenne, France). As per the protocol, initially, the dough was held at 30 °C for 8 min in the equipment mixer (Figure 2). In the next stage, the dough temperature was increased to 90 °C and maintained at that for 8 min. This stage enables the gelatinization of starch granules. Furthermore, the temperature was reduced to 50 °C to simulate starch retrogradation. The experiments evaluated protein properties related to stability, elasticity, and weakening. Starch gelatinization and retrogradation as affected by mixing and temperature were also monitored. The parameters measured during the Mixolab analysis are C1 (initial consistency or torque during mixing at 30 °C (Nm)), C2 (minimum torque (Nm) representing protein weakening), C3 (peak torque experienced upon heating corresponding to starch gelatinization), C4 (minimum torque during the heating phase representing gel stability), and C5 (torque during the cooling stage at 50 °C (Nm)) corresponding to starch retrogradation during the cooling phase. The slopes α, β, and γ measure the protein breakdown rate under the influence of heat, the rate of gelatinization, and the cooking stability rate, respectively. Apart from the primary parameters measured from the Mixolab analysis, the derived parameters evaluated included the protein weakening range (C2–C1), the pasting range (C3–C2), the cooking stability (C4–C3), the cooling setback (C5–C4), and the pasting temperature range (D3–D2). Additionally, we measured the dough development time, i.e., the time to reach 1.1 Nm torque; the targeted consistency for bread dough (T1 in minutes); and the mixing resistance of the dough, represented as stability (DS in minutes). It should be noted that the flour yield from 3 BK was insufficient for Mixolab analysis and was excluded for all treatment combinations.

### 2.6. Statistical Analysis

The effect of tempering time, moisture, samples, and fraction was assessed with a fixed-effect model, as described by [31] using SAS Studio (SAS Institute Inc., Cary, NC, USA). Analysis of variance (ANOVA) was used to test the significant differences in the chemical and rheological qualities of the obtained flour. The homogeneity of variances was analyzed using the Levene test. Tukey’s test was performed following ANOVA to compare the means at a significance level of 5%. Principal component analysis (PCA) was applied to the data for pattern recognition. The data consisted of 5 rows (wheat) × 14 columns (quality indicators) for the samples. In the PCA, the number of components to be retained was determined based on the eigenvalues (one criterion) or Kaiser criterion.

## 3. Results

### 3.1. Flour Yield

In the study, HRW wheat samples had a higher milling yield (73.2%) than HRS (68.4%). Additionally, the incorporation of greater percentages of HRS in the blend decreased the milling yield. For instance, B1, B2, and B3 had flour yields of 74.3%, 73.3%, and 72.9%, respectively (Table 1). It should be noted that although there was a reduction in flour yield with an increase in HRS content, the decrease was not statistically significant (*p* > 0.05). However, in commercial milling, profitability is largely dependent on extraction. Thus, while not statistically significant, depending on the capacity of mills, even a 0.5% increase or decrease in the flour yield over time can influence the commercial value. Furthermore, varying the tempering moisture content from 14% to 18% resulted in an increase in flour yield from 71.7% to 73.1%. On the other hand, longer tempering time significantly (*p* < 0.05) reduced the flour yield. For instance, a yield as high as 73.7% was obtained by tempering the grains for 16 h, while it was reduced to 70.6% at 24 h (Tables S1 and S2).

### 3.2. Compositional Analysis

The protein, ash, and damaged starch content of the flour samples varied with respect to the milling stream and tempering conditions. The influence of blending was more prominent on the protein content of the flour (Table 1 and Table 2) than on ash and damaged starch. Increasing the HRS content in the blend varied the protein content of B1, B2, and B3 to 10.3, 11.4, and 12.9%, respectively (Table 1). Additionally, there was a significant increase in the protein content (*p* < 0.05) with successive passages in the break stream. It varied from 13.41% in 1 BK to 16.75% in 3 BK. Tempering conditions also significantly (*p* < 0.05) affected the protein content of the milling streams (Table 2). Similar to the findings of [10], in our study, increasing the tempering moisture content reduced the protein content of the analyzed flour. Similar variations were also observed in the ash content of the flour. The ash content increased from 0.47% to 1.16% in the reduction streams (*p* < 0.05). Similar increases in the ash content of reduction streams were reported by [32]. Apart from the milling streams, blending and the tempering time significantly affected the ash content of the flour. Tempering the wheat for a shorter duration (16 h) decreased the ash content to 0.47%. Although the tempering time had a greater influence than moisture on the ash content (Table 2), the tempering moisture also significantly affected the ash content of flour. The ash content was lowest (0.49%) when samples were tempered to 18%, whereas it increased to 0.52% and 0.51% when the moisture was 14% and 16% (Tables S1 and S2).

The degree of starch damage varied significantly (*p* < 0.05) in the milling streams (Table S3). In the break stream, the damaged starch content varied from 4.97% to 6.13%, while in the reduction stream, damage starch as high as 14.73% was observed. In the reduction streams of the mill, the degree of crushing was higher due to a smaller roll gap, resulting in finer flour with higher damaged starch. Increasing the tempering moisture from 14% to 18% resulted in an increase in the damaged starch content from 8.7% to 9.5%. When increasing the tempering time from 16 to 24 h, the damaged starch content decreased from 9.37% to 8.83%. Although there was a decrease in the damaged starch content, the reduction was not statistically significant (*p* > 0.05). ANOVA results showed that the interaction between moisture content and time had a significant impact on the damaged starch content of the flours (Table 2). Additionally, the data for damaged starch showed that the tempering time influenced the variations in break flour streams, whereas the tempering moisture had a prominent impact on reduction streams. Higher percentages of HRS in the blends increased the damaged starch content of the flour (Table 1), although the differences were not statistically significant (*p* > 0.05). Moreover, ANOVA results showed that milling streams (fraction) and tempering conditions significantly contributed to the variation in damaged starch, rather than blending.

### 3.3. Particle Size Analysis

The particle characteristics of flour were represented by *d10*, *d50*, and *d90* values. Differences in grain hardness between HRW and HRS samples contributed to variations in the *d10*, *d50*, and *d90* values of the samples. For example, the *d10* values for HRW and HRS were 22.6 µm and 25.4 µm, respectively. Blending significantly influenced the particle characteristics of flour (Table 1 and Table 2). Additionally, differences were observed in the diameters among the mill streams (fractions). For instance, *d10*, *d50*, and *d90* for flour from 1 BK were 40.7, 96.6, and 166.8 µm, respectively, whereas it was 20.1, 60.7, and 122.7 µm, respectively, for the flour obtained from 3 M. Differences in operational parameters such as roll gap and roll differential influenced the particle size distribution of the milling streams. While there were significant (*p* < 0.05) changes in the *d10* value with respect to tempering moisture (Table S1), the *d50* and *d90* were not significantly (*p* > 0.05) affected by tempering conditions. Apart from blending and moisture content, the interactions between wheat × tempering time and moisture content × tempering time also contributed to the variations in particle characteristics such as *d10*, *d50*, and *d90* (Table 2).

### 3.4. Thermomechanical Analysis

The rheological behavior of wheat dough is influenced by water, starch, and protein. In addition to its major components, other minor constituents such as pentosans and enzymes contribute to the complex rheological characteristics of the flour. When wheat flour is mixed with sufficient water, solubilization and unfolding of the protein occur, and it forms a continuous network. The starch molecules are embedded into this protein network to form a viscoelastic dough. The viscoelastic characteristics of dough undergo changes during mixing and heating. Mixolab measures the changes in the dough system pertaining to simultaneous heating and mixing. Water absorption (WA) is the amount of water required for the samples to reach an initial torque of 1.1 Nm (at 30 °C) for dough development. In the analysis, WA varied with respect to samples and milling stream. HRS samples had the highest WA of 68.8%. WA increased from 58.3% to 72.2% as the samples varied from 1 BK to 3 M. Even though grain moistening to 18% and longer tempering duration (24 h) increased the WA, the changes were statistically not significant (*p* > 0.05).

The C1 scores of the Mixolab analysis (corresponding to dough development at 30 °C) were statistically similar with respect to tempering conditions, sample, and milling streams. Although there was a gradual increase in the values with an increase in the protein content of the flour for the blends, the increase was statistically insignificant (*p* > 0.05). T1 is the time required by the equipment to reach the required consistency of 1.1 Nm. T1 for the flour was higher (155.29 s) when the grains were tempered to 14% moisture content compared with 18% (135.75 s) (Table 3). Stability (DS) represents the time for which the torque of the dough stayed above 1.1 Nm during the initial mixing phase, and it varied from 6.9 min to 9.3 min for different samples. It also varied significantly (*p* < 0.05) with the milling stream. For instance, DS decreased from 10.1 min (1 BK) to 8.2 min for 3 M. Although there was an increase in dough stability with grain moistening and tempering time, the increase was statistically insignificant (*p* > 0.05).

Increasing the temperature of the system to 60 °C weakened the proteins and initiated the gelatinization of the dough. The low-energy hydrogen bonds in the glutenin network were easily broken with an increase in temperature [30]. Thus, in the second phase of the Mixolab analysis, the torque value (C2) decreased. There was a significant increase in the C2 value with an increase in the tempering moisture content. C2 values also decreased with flour from the successive milling streams (0.48 to 0.38). The protein weakening temperature (D2) varied among the samples. The weakening temperature was 55 °C for HRS, whereas it was 53.6 °C for HRW (Table 3). The protein of the break flour streams had comparatively higher thermal stability (55.3 °C) than those from the reduction streams (~54 °C). Additionally, tempering the grains to 18% decreased the thermal stability (with mixing) to 53.5 °C compared with the 55 °C observed when tempering to 14%. Furthermore, the protein breakdown rate (α) was statistically similar for all the test conditions. The tempering conditions and blending did not significantly vary the protein breakdown rate.

In the third stage of testing, the C3 scores (indicating starch gelatinization and pasting) decreased with blending. The decrease was proportional to the increase in HRS content. Among the samples, the lowest C3 value was observed for the HRS wheat (0.9 Nm). Tempering conditions did not significantly (*p* > 0.05) influence the starch gelatinization and pasting characteristics (C3 values). The flour from the reduction streams had significantly lower C3 than the break streams. Additionally, for the break streams, C3 values were as high as 1.5 Nm, whereas it was successively reduced to 1.3, 1.1, and 0.8 Nm with the passage from 1 M to 3 M. The rate of gelatinization (β) was statistically different among the samples (Table 3). For example, β varied from 0.09 (HRS) to 0.21 (HRW). Tempering conditions had a negligible influence on the rate of gelatinization.

In the last stage of analysis, using Mixolab, we measured gel stability (C4), i.e., the soundness of the starch fraction of the flour. It also corresponds to the presence of amylases in the samples. Although the variation was not significant (*p* > 0.05), C4 varied with respect to the wheat samples (ranging from 1.4 to 1.7 Nm). Additionally, C4 values varied greatly among the milling streams. C4 was 1.8 Nm for 1 BK, whereas it was reduced to 1.2 Nm for 3 M. The cooking stability rate (γ) of the analyzed samples was not influenced by blending and tempering conditions. The torque obtained at the end of the test (C5) varied significantly (*p* < 0.05) with respect to the samples. The final torque was 2.6 Nm for B1 and decreased to 1.9 Nm for HRS samples. The flour samples exhibited a decrease in the final torque with an increase in protein content. Even though the break streams had higher torque than the reduction streams, there was a significant reduction in C5 torque with successive milling streams.

The blending and tempering moisture had a significant (*p* < 0.05) influence on the pasting temperature range (D3–D2), which is the difference between the protein weakening temperature and the temperature at C3. For instance, it increased from 14.71 °C for the samples tempered to 14% (w.b) to 17.84 °C for those tempered to 18% (w.b). Increasing the proportion of the HRS content significantly (*p* < 0.05) narrowed the pasting temperature range (Table 3).

### 3.5. Principal Component Analysis (PCA)

**Figure 3:**
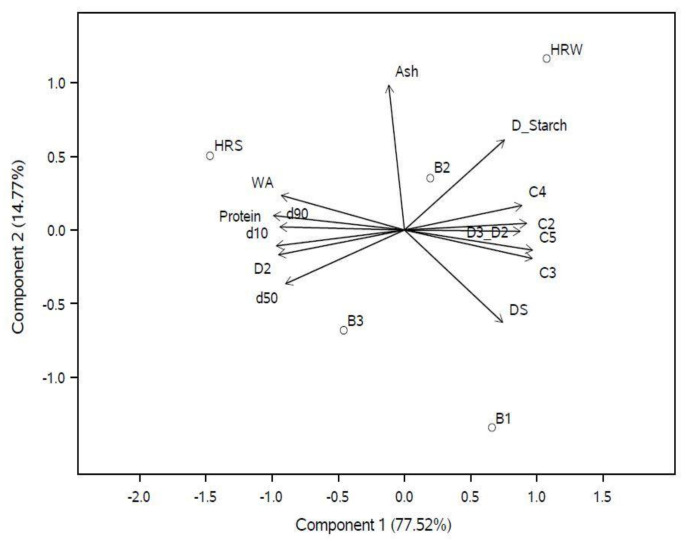
PCA biplot (PC1 vs. PC2) for physicochemical and rheological properties. HRW: hard red winter; HRS: hard red spring; B1: Blend 1; B2: Blend 2; B3: Blend 3; D_starch: damaged starch; WA: water absorption; C2: protein weakening; C3: starch gelatinization; C4: starch gelling; C5: starch retrogradation; DS: dough stability; D2: temperature at protein weakening; D3_D2: pasting temperature range (D3–D2).

PCA was performed to understand the quality variation among the blended samples and their interrelationships. In the process, the original variables were compressed into two principal components (eigenvalue greater than 1). This enables the identification of similarities and differences based on the correlation of variables and pattern recognition. In the analysis, 92.29% of the variability in the data was explained by the first two PCs, where 77.5% of the variability was explained by PC1 and 14.8% by PC2 (Figure 2). In PCA plots, the variables appearing close to each other represent positive correlations, whereas those in the opposite directions indicate negative correlations. Upon analyzing the PCA biplot, it was found that an increase in WA was positively correlated with protein content (r = 0.95), ash content (r = 0.35), and particle characteristics than damaged starch. The damaged starch content influenced the C3, C4, and C5 values (r = 0.58, 0.58, and 0.87, respectively) of the flour. Dough stability (DS) (r = 0.2) and the pasting temperature range (D3–D2) (r = 0.57) were positively correlated with the damaged starch while negatively correlated with the protein content (r = −0.81 and −0.90, respectively). Additionally, a positive correlation between protein and the protein weakening temperature (D2) (r = 0.95) as well as WA (r = 0.95) was observed. As expected, Blend B2 (with 50% HRW and HRS) had rheological characteristics in between the control samples (Figure 3). Rheological characteristics, especially starch gelatinization and pasting, gel stability, and final viscosity, were mostly determined by the similarity of B1 with HRW.

## 4. Discussion

### 4.1. Compositional Analysis

In the analysis, there was an increase in the protein and damaged starch content of the flour with the milling stream and blends. It was observed that the increase in the protein content was prominent in the break stream, while the damaged starch content varied greatly in the reduction streams. This can be associated with the differences in the protein distribution inside the wheat kernels. In a typical wheat grain, the proportion of protein in the mature endosperm increases radially towards the outward direction [33]. This differential distribution of the protein inside the kernel, the presence of peripheral endosperm, and the inclusion of finer bran particles rich in protein [19,34] contribute to the increase in the protein content of the flour in the break streams. Along with protein content, the protein composition also varies throughout the wheat kernel, contributing to variability in functional properties. Thus, flour from the central endosperm has better functional properties, even though it has lower protein content [34]. There was a higher percentage of ash content in the tail-end streams [32], as the final milling streams receive relatively bran-rich tail-end flour from the 3 BK. Furthermore, at lower moisture levels, the kernels are brittle, resulting in finer bran fractions in the flour, which contribute to higher ash contents.

Mechanical damage in starch is inevitable during the milling of grains [35]. Shear forces during roller milling of wheat cause crystalline damage in the starch–protein interface, resulting in mechanical damage in starch [36]. The extent of damage is influenced by kernel characteristics (hardness) and mill operational parameters (roll gap, roll speed, and roll differential). Furthermore, milling intensity increases in the reduction streams with a reduced roll gap, resulting in finer flour fractions. As the flour fineness increases, the degree of starch damage also increases [37,38,39]. Tempering conditions also influence the composition of the flour [10]. Tempering, or grain moistening, results in the development of cracks in the endosperm [40]. The plane of these cracks intersects transversely with the starch granules [10]. This increases the chances of mechanical damage to starch with an increase in tempering moisture [41].

### 4.2. Particle Size

The particle size distributiosn of flour depends on factors such as tempering, roll adjustments, and wheat characteristics [42]. As expected, the particle size of the flour varied with respect to tempering conditions. Higher mean particle sizes were observed when the grains were tempered to 18% [10,31]. In the study, the tempering time did not impact the *d10* values, as the studied time (greater than 16 h) was significant enough to ensure the moisture migration inside the kernels. Moreover, there was an increase in the mean particle size of the flour with an increasse in the proportion of HRS in the blend. With a higher proportion of HRS, the overall hardness of the wheat kernels increased, resulting in larger flour particles [43]. The particle size of the flour significantly influenced the physicochemical and functional properties of the wheat flour [44].

### 4.3. Thermomechanical Properties

The WA of the flour depends on the quality and content of proteins, the native starch, the damaged starch content, and the fiber (pentosans) content [30]. In the study, the protein content varied among the samples and was a prominent contributor to the WA (Figure 3). A higher percentage of HRS concurrently increased the WA due to the greater content of gliadins and glutenins [45]. Moreover, the increases were consistent with the observations of [46] (Table 3). According to the empirical method proposed by Sluimer [46], WA is expected to increase by 1% (*w*/*w*) for every 1% increase in protein content [30]. Additionally, the proportion of B-type granules is higher in the HRS wheat [47], and these granules have a higher affinity for water than A-type granules at room temperature [48], thus increasing the WA with HRS content in the samples.

During the mixing stage, the hydration of proteins leads to their expansion. This increases the interaction between the thiol groups of the protein, and disulfide bonds are created, which plays a significant role in the formation of the gluten network. Furthermore, the starch granules are embedded in the gluten network forming the dough. Thus, the behavior of the dough is also dependent on the starch content. Increasing the temperature from 30 to 90 °C during the analysis weakened the protein network and gelatinized the starch. Unlike in batter, where the complete gelatinization of the starch occurs upon heating, in dough systems, the gelatinization of the starch is incomplete due to the limited availability of water. This leads to the transfer of moisture from proteins to starch during cooking [30]. These changes in the protein and starch during mixing and cooking depend on the composition of starch (A- and B-type granules) [47] and protein (gliadin, glutenin, and polymeric proteins) [45], temperature, and water activity [49]. The observed differences in the Mixolab parameters (torque C2 to C5) of the samples can be attributed to these compositional differences in HRW and HRS.

The distribution of lipids [50], fatty acids [20], free amino acids and sulfur [51], free thiol groups [52], disulfide linkages [36], and enzymes [21] varies among different mill streams. Additionally, there is a variable distribution of protein concentration and composition [19,34,53] among different mill streams, contributing to the differences in the rheological properties. The concentration of polymeric protein in relation to monomeric protein is greater in break streams than in reduction streams [53], resulting in a lower degree of disulfide cross-linking [19,34,53]. These differences result in higher C2 values of the break flour streams than in reduction streams. Furthermore, reduction streams have higher α-amylase content [21] and damaged starch. It is reported that damaged starch is prone to hydrolysis by α-amylase [54]. The concomitant increase in the enzyme and damaged starch is associated with a decrease in C3, C4, and C5 torques [55] in reduction streams. Moreover, the activity of the enzyme leads to the production of dextrins, which in turn impacts the water-holding capacity and porosity of the dough [56]. The difference in these distributions corresponds to variability in the rheological properties among milling streams.

PCA results revealed a negative correlation between particle characteristics and damaged starch. This inverse relationship implies that a reduction in particle size was associated with an increase in starch damage, which in turn was positively correlated with WA. Moreover, a reduction in particle size promotes the redox conversion of sulfhydryl (-SH) groups to disulfide groups [36]. This leads to the cross-linking of low-molecular-weight gluten to form high-molecular-weight glutenin subunits, thereby increasing the gluten macro-molecular protein content [36]. Ash content also influenced WA. This is attributed to the higher water-binding capacity of the arabinoxylans present in the aleurone layer and bran. Thus, the particle characteristics, ash, and protein content interfere with water absorption characteristics [19,34]. Damaged starch influenced the gelatinization and pasting properties of the dough [57]. An increase in gliadin content with the incorporation of HRS in the blends decreased the stability of the dough [37,45]. This resulted in a negative correlation between protein content and dough stability.

By blending HRS and HRW, the protein content was intentionally varied, contributing to a change in the quality of protein among the samples. Subsequent changes in the starch granule type and distribution also occurred in the blends [47]. These differences contribute to changes in WA and other rheological properties of the dough. From the PCA results, it is evident that the WA characteristics were closely associated with the protein content rather than the damaged starch. Thus, in blends with a higher proportion of HRS, the protein content was the primary determinant of particle characteristics, WA, and pasting temperature.

## 5. Conclusions

In summary, tempering and blending affected the composition and particle characteristics of flour. These variables had a significant influence on particle size, damaged starch content, and protein content; subsequent differences were observed in rheological properties. The flour from break and reduction streams differed in composition and rheological properties, contributing to the differences in functionality. Variations in the properties were also exhibited with successive milling streams in both break and reduction streams. An increased proportion of HRS in the blends varied the pasting characteristics and cooking stability of the flour. The experiments illustrated that blending not only affects the protein content and composition but also the starch quality and thus rheological properties. Therefore, tempering and blending can effectively modify the rheological and functional properties of flour.

## Figures and Tables

**Table 1:** Effect of hard wheat blending on flour yield, particle characteristics, and chemical properties of straight-grade wheat flour.

	Flour Yield	*d10*	*d50*	*d90*	Protein	Damaged Starch	Ash
Sample	(%)	(μm)	(μm)	(μm)	(%)	(%)	(%)
HRW	73.22 (3.6) ^A^	22.61 (0.96) ^B^	69.03 (10.1) ^A^	131.82 (17.9) ^A^	9.41 (0.42) ^D^	9.25 (2.32) ^A^	0.55 (0.12) ^A^
B1	74.33 (2.9) ^A^	23.43 (2.55) ^AB^	74.24 (13.4) ^A^	130.14 (20.6) ^A^	10.28 (0.66) ^CD^	8.37 (1.26) ^A^	0.44 (0.11) ^A^
B2	73.23 (2.7) ^A^	24.09 (1.80) ^AB^	75.09 (7.1) ^A^	141.01 (13.8) ^A^	11.39 (1.11) ^C^	9.51 (1.89) ^A^	0.44 (0.21) ^A^
B3	72.88 (1.9) ^A^	24.00 (2.30) ^AB^	77.76 (6.6) ^A^	129.99 (7.7) ^A^	12.94 (1.43) ^B^	9.08 (2.03) ^A^	0.43 (0.18) ^A^
HRS	68.38 (7.6) ^A^	25.44 (3.01) ^A^	79.55 (9.9) ^A^	143.02 (10.0) ^A^	15.88 (0.69) ^A^	8.63 (2.32) ^A^	0.60 (0.20) ^A^

The values are represented as mean (±standard deviation). Values designated by different letters in a column are significantly different at *p* = 0.05. HRW: hard red winter; HRS: hard red spring; B1: Blend 1 (75% HRW:25% HRS); B2: Blend 2 (50% HRW:50% HRS); B3: Blend 3 (25% HRW:75% HRS).

**Table 2:** Percent variance components across the dataset for physicochemical characteristics as a function of wheat, moisture content (MC), tempering time, milling stream, and their interactions.

Source	Damaged Starch	Ash	Protein	*d10*	*d50*	*d90*
Wheat	3.32	8.62	67.82	7.74	9.35	5.76
Moisture content (MC)	4.4	1.91	0.5	ns	4.74	ns
Time	2.68	7.9	0.91	ns	ns	ns
Milling stream	77.75	64.92	26.89	82.95	63.88	58.34
Wheat × MC	0.86	4.78	0.94	ns	ns	ns
Wheat × Time	1.68	1.19	0.38	0.94	1.95	2.21
Wheat × Milling stream	ns	ns	0.57	0.8	ns	1.86
MC × Time	5.91	4.52	0.61	2.88	8.36	5.75
MC × Milling stream	ns	ns	0.25	1.04	1.98	ns
Time × Milling stream	ns	0.87	0.2	ns	ns	ns
Wheat × MC × Time	1.86	2.96	0.53	ns	1.55	ns
Wheat × MC × Milling stream	ns	ns	ns	ns	1.21	1.98
Wheat × Time × Milling stream	ns	ns	0.16	ns	ns	1.95
MC × Time × Milling stream	0.37	ns	ns	ns	1.88	1.69

ns: not significant.

**Table 3:** Effect of hard wheat blending on rheological properties of dough measured using Mixolab.

Rheological Properties	Samples				
	HRW	B1	B2	B3	HRS
WA (%)	62.9 (6.4) ^B^	61.5 (6.0) ^B^	62.8 (3.4) ^B^	65.9 (5.2) ^AB^	68.9 (3.1) ^A^
C2 (Nm)	0.48 (0.05) ^A^	0.46 (0.05) ^A^	0.47 (0.04) ^A^	0.45 (0.05) ^A^	0.4 (0.04) ^B^
C3 (Nm)	1.5 (0.5) ^A^	1.5 (0.5) ^A^	1.3 (0.4) ^A^	1.2 (0.4) ^AB^	0.9 (0.3) ^B^
C4 (Nm)	1.6 (0.3) ^A^	1.6 (0.3) ^A^	1.7 (0.4) ^A^	1.4 (0.5) ^A^	1.4 (0.2) ^A^
C5 (Nm)	2.5 (0.6) ^A^	2.6 (0.5) ^A^	2.5 (0.3) ^A^	2.2 (0.5) ^AB^	1.9 (0.3) ^B^
α	−0.08 (0.0) ^A^	−0.11 (0.2) ^A^	−0.08 (0.0) ^A^	−0.07 (0.0) ^A^	−0.08 (0.0) ^A^
β	0.21 (0.1) ^A^	0.14 (0.1) ^AB^	0.15 (0.1) ^AB^	0.12 (0.1) ^B^	0.09 (0.1) ^B^
γ	−0.03 (0.1) ^AB^	−0.06(0.1) ^B^	0.02 (0.1) ^A^	−0.02 (0.1) ^AB^	0.03 (0.1) ^A^
C2–C1 (Nm)	−0.63 (0.0) ^A^	−0.64 (0.1) ^A^	−0.65 (0.0) ^A^	−0.61 (0.2) ^A^	−0.71 (0.0) ^B^
C3–C2 (Nm)	0.96 (0.5) ^A^	1.04 (0.1) ^A^	0.8 (0.4) ^AB^	0.82 (0.3) ^AB^	0.51 (0.3) ^B^
C4–C3 (Nm)	0.1 (0.4) ^B^	0.1 (0.5) ^B^	0.4 (0.5) ^AB^	0.2 (0.4) ^AB^	0.5 (0.2) ^A^
C5–C4 (Nm)	0.91 (0.3) ^A^	0.99 (0.5) ^A^	0.8 (0.34) ^AB^	0.77 (0.4) ^AB^	0.55 (0.1) ^B^
T1 (s)	66.68 (43.7) ^B^	146.2 (143.3) ^AB^	165.6 (135.6) ^AB^	212.1 (164.5) ^A^	184 (88.6) ^A^
Stability (min)	9.02 (0.7) ^B^	9.91 (1.4) ^A^	9.0 (1.0) ^B^	8.86 (0.8) ^B^	8.30 (0.8) ^B^
D2 (°C)	53.59 (2.0) ^B^	54.02 (1.0) ^AB^	54.43 (1.3) ^AB^	54.82 (1.4) ^AB^	55.03 (1.3) ^A^
D3–D2 (°C)	19.59 (4.9) ^A^	18.39 (5.3) ^AB^	13.95 (4.3) ^C^	14.21 (5.2) ^BC^	12.87 (3.5) ^C^

Values are represented as mean (±standard deviation). Values designated by different letters in a row are significantly different at *p* = 0.05. HRW: hard red winter; HRS: hard red spring; B1: Blend 1 (25% HRS); B2: Blend 2 (50% HRS); B3: Blend 3 (75% HRS); WA: water absorption; C1: torque at mixing (30 °C); C2: protein weakening; C3: starch gelatinization; C4: starch gelling; C5: starch retrogradation; α: protein breakdown rate; β: rate of gelatinization; γ: cooking stability rate; C2–C1: protein weakening range; C3–C2: pasting range; C4–C3: cooking stability; C5–C4: cooling setback, T1: time to reach C1; DS: stability; D2: the temperature at protein weakening; D3–D2: pasting temperature range.

## Data Availability

The data are contained within the article and supplementary material.

## Supplementary Materials

The following supporting information can be downloaded at: https://www.mdpi.com/article/10.3390/foods12102078/s1, Table S1: Effect of tempering moisture on particle characteristics and chemical properties of straight-grade flour of wheat blends; Table S2: Effect of tempering time on particle characteristics and chemical properties of straight-grade flour of wheat blends. Table S3: Variation in particle characteristics and chemical properties of flour obtained from different milling streams (fraction).
