# Supplementary material for: Investigating the Contribution of Blending on the Dough Rheology of Roller-Milled Hard Red Wheat
Source: Foods. 2023 May 22;12(10):2078. doi: 10.3390/foods12102078 (PMC10217494; doi:10.3390/foods12102078)
Supplement: Supplementary file 1 [file foods-12-02078-s001.zip › foods-2376629-supplementary.pdf]

Supplemental data

Table S1. Effect of tempering moisture on particle characteristics and chemical properties of straight grade flours of wheat blends

**Tempering moisture (%)**	**Sample**	**Flour yield (%)**	***d10* (μm)**	***d50* (μm)**	***d90* (μm)**	**Protein (%)**	**Damaged starch (%)**	**Ash (%)**
14	HRW	69.4 (4.0)	22.93 (1.1)	75.57 (14.7)	139.36 (13.4)	9.72 (0.3)	7.98 (1.1)	0.53 (0.10)
	B1	75.5 (1.3)	24.69 (2.6)	79.26 (11.6)	127.18 (12.0)	10.74 (0.6)	9.07 (1.4)	0.51 (0.07)
	B2	72.6 (1.6)	22.55 (1.7)	69.45 (6.0)	135.95 (18.1)	12.81 (0.4)	8.07 (2.8)	0.54 (0.05)
	B3	73.2 (0.7)	25.88 (2.7)	76.09 (6.8)	124.17 (4.9)	13.31 (1.4)	9.09 (1.8)	0.48 (0.08)
	HRS	67.9 (4.2)	28.15 (3.1)	83.82 (4.9)	146.53 (1.8)	16.14 (1.3)	9.90 (2.6)	0.50 (0.16)
16	HRW	76.9 (4.0)	22.30 (1.1)	61.17 (4.6)	127.60 (3.9)	9.11 (0.5)	8.10 (0.9)	0.49 (0.11)
	B1	73.6 (2.2)	23.95 (2.1)	79.82 (9.4)	129.45 (3.1)	10.89 (0.3)	8.42 (1.1)	0.47 (0.09)
	B2	72.8 (2.3)	25.26 (0.7)	81.04 (6.8)	151.17 (13.6)	11.40 (0.7)	9.86 (1.8)	0.45 (0.03)
	B3	73.3 (3.4)	23.40 (0.4)	81.95 (5.9)	132.17 (5.0)	13.34 (1.8)	8.79 (3.0)	0.56 (0.23)
	HRS	72.7 (2.8)	24.82 (2.3)	83.68 (13.1)	150.57 (7.8)	15.68 (0.3)	10.32 (2.8)	0.54 (0.11)
18	HRW	74.0 (6.1)	22.62 (1.0)	70.34 (3.2)	128.49 (4.0)	9.40 (0.3)	8.89 (1.7)	0.53 (0.20)
	B1	73.9 (5.0)	21.66 (2.6)	63.62 (5.5)	133.80 (8.8)	9.68 (0.9)	9.64 (1.6)	0.46 (0.12)
	B2	74.4 (4.2)	24.46 (1.9)	74.76 (4.5)	135.90 (3.8)	11.42 (0.6)	10.11 (1.5)	0.51 (0.17)
	B3	72.2 (1.3)	22.74 (2.4)	75.24 (7.2)	133.65 (10.6)	12.17 (1.3)	9.37 (2.1)	0.49 (0.04)
	HRS	71.2 (2.0)	23.35 (1.7)	71.13 (6.1)	131.97 (7.0)	15.82 (0.3)	9.46 (3.7)	0.59 (0.08)

The values are represented as mean (± standard deviation). HRW: Hard red winter, HRS: Hard red spring, B1: Blend 1 (75% HRW: 25% HRS), B2: Blend 2 (50% HRW: 50% HRS), B3: Blend 3 (25% HRW: 75% HRS)

Table S2. Effect of tempering time on particle characteristics and chemical properties of straight grade flours of wheat blends

Tempering time (h)		Flour Yield	*d10*	*d50*	*d90*	Protein	Damaged starch	Ash
	Sample	(%)	(μm)	(μm)	(μm)	(%)	(%)	(%)
16	HRW	75.4 (3.5)	21.96 (0.8)	66.38 (8.7)	132.87 (13.3)	9.45 (0.4)	9.05 (0.7)	0.48 (0.14)
	B1	74.5 (3.1)	23.66 (2.3)	79.18 (12.1)	120.47 (7.9)	10.22 (0.9)	8.35 (2.0)	0.50 (0.09)
	B2	75.5 (3.1)	23.51 (2.4)	77.70 (4.5)	141.82 (13.1)	11.52 (0.8)	10.37 (1.6)	0.39 (0.03)
	B3	71.9 (0.9)	23.10 (0.5)	77.52 (7.1)	131.55 (12.3)	14.33 (0.8)	10.35 (1.4)	0.45 (0.1)
	HRS	71.4 (2.4)	24.38 (1.1)	75.22 (3.1)	144.27 (6.1)	16.22 (1.2)	9.07 (2.5)	0.52 (0.14)
20	HRW	76.4 (3.0)	22.96 (0.7)	65.96 (6.2)	117.61 (8.0)	9.30 (0.4)	8.61 (0.5)	0.56 (0.06)
	B1	75.7 (1.5)	22.57 (4.3)	68.73 (7.0)	143.23 (13.4)	10.30 (0.3)	8.64 (1.0)	0.46 (0.11)
	B2	73.6 (1.4)	24.37 (2.3)	71.63 (8.1)	137.48 (15.4)	11.72 (0.8)	9.82 (0.5)	0.50 (0.06)
	B3	74.5 (2.3)	24.59 (1.0)	76.77 (3.8)	128.47 (5.1)	12.63 (2.2)	10.09 (1.5)	0.57 (0.21)
	HRS	71.6 (1.1)	26.31 (2.3)	80.86 (6.7)	141.18 (7.0)	15.92 (0.3)	9.29 (1.7)	0.55 (0.1)
24	HRW	68.5 (4.0)	22.93 (1.3)	74.75 (4.9)	144.98 (4.6)	9.47 (0.6)	10.09 (2.1)	0.61 (0.16)
	B1	72.8 (4.1)	24.06 (1.0)	74.81 (4.0)	126.73 (4.9)	9.78 (1.3)	9.14 (2.0)	0.47 (0.09)
	B2	70.7 (0.4)	24.40 (1.0)	75.93 (9.4)	143.72 (8.0)	11.84 (1.2)	8.61 (3.0)	0.56 (0.12)
	B3	72.3 (1.6)	24.32 (4.2)	78.99 (10.3)	129.97 (7.3)	13.85 (0.9)	6.81 (0.8)	0.52 (0.07)
	HRS	68.8 (5.2)	25.63 (5.2)	82.56 (7.1)	143.62 (7.5)	15.49 (0.3)	9.52 (2.9)	0.55 (0.14)

The values are represented as mean (± standard deviation). HRW: Hard red winter, HRS: Hard red spring, B1: Blend 1 (75% HRW: 25% HRS), B2: Blend 2 (50% HRW: 50% HRS), B3: Blend 3 (25% HRW: 75% HRS)

Table S3. Variation in particle characteristics and chemical properties of flour obtained from different milling streams (fraction)

**Milling fraction**	***d10*** (μm)	***d50*** (μm)	***d90*** (μm)	**Protein** (%)	**Damaged starch** (%)	**Ash** (%)
1BK	40.78 (7.7)	96.89 (10.6)	167.19 (13.1)	13.47 (2.3)	4.99 (1.3)	0.45 (0.2)
2BK	39.02 (6.2)	74.61 (8.7)	144.56 (8.9)	15.01 (3.0)	5.44 (1.4)	0.45(0.2)
3BK	31.76 (4.6)	79.89 (6.2)	128.97 (9.4)	16.89 (3.3)	6.05 (1.6)	0.64 (0.2)
1M	24.78 (3.7)	74.61 (11.2)	134.10 (8.4)	11.73 (2.4)	8.82 (2.0)	0.47 (0.2)
2M	22.45 (3.2)	69.05 (6.2)	128.11 (5.8)	11.31 (2.5)	12.26 (2.7)	0.71 (0.2)
3M	20.00 (2.0)	60.05 (13.7)	122.2 (8.8)	12.58 (2.2)	14.92 (2.6)	1.17 (0.2)

The values are represented as mean (± standard deviation). 1BK: 1st break roll, 2BK: 2nd break roll, 3BK: 3rd break roll, 1M: 1st reduction roll, 2M: 2nd reduction roll, 3M: 3rd reduction roll.
